# Geographical and Temporal Body Size Variation in a Reptile: Roles of Sex, Ecology, Phylogeny and Ecology Structured in Phylogeny

**DOI:** 10.1371/journal.pone.0104026

**Published:** 2014-08-04

**Authors:** Pedro Aragón, Patrick S. Fitze

**Affiliations:** 1 Departamento de Biogeografía y Cambio Global, Museo Nacional de Ciencias Naturales, Madrid, Spain; 2 Departamento de Biodiversidad y Biología Evolutiva, Museo Nacional de Ciencias Naturales, Madrid, Spain; 3 Université de Lausanne, Department of Ecology and Evolution (DEE), Biophore, Lausanne, Switzerland; 4 Instituto Pirenaico de Ecología (IPE-CSIC), Avenida Regimiento de Galicia s/n, Jaca, Spain; 5 Fundación Araid, Edificio Pignatelli, Zaragoza, Spain; Université de Sherbrooke, Canada

## Abstract

Geographical body size variation has long interested evolutionary biologists, and a range of mechanisms have been proposed to explain the observed patterns. It is considered to be more puzzling in ectotherms than in endotherms, and integrative approaches are necessary for testing non-exclusive alternative mechanisms. Using lacertid lizards as a model, we adopted an integrative approach, testing different hypotheses for both sexes while incorporating temporal, spatial, and phylogenetic autocorrelation at the individual level. We used data on the Spanish Sand Racer species group from a field survey to disentangle different sources of body size variation through environmental and individual genetic data, while accounting for temporal and spatial autocorrelation. A variation partitioning method was applied to separate independent and shared components of ecology and phylogeny, and estimated their significance. Then, we fed-back our models by controlling for relevant independent components. The pattern was consistent with the geographical Bergmann's cline and the experimental temperature-size rule: adults were larger at lower temperatures (and/or higher elevations). This result was confirmed with additional multi-year independent data-set derived from the literature. Variation partitioning showed no sex differences in phylogenetic inertia but showed sex differences in the independent component of ecology; primarily due to growth differences. Interestingly, only after controlling for independent components did primary productivity also emerge as an important predictor explaining size variation in both sexes. This study highlights the importance of integrating individual-based genetic information, relevant ecological parameters, and temporal and spatial autocorrelation in sex-specific models to detect potentially important hidden effects. Our individual-based approach devoted to extract and control for independent components was useful to reveal hidden effects linked with alternative non-exclusive hypothesis, such as those of primary productivity. Also, including measurement date allowed disentangling and controlling for short-term temporal autocorrelation reflecting sex-specific growth plasticity.

## Introduction

Spatial and temporal variation in phenotypic traits is a central issue in ecology and evolution. Geographical variation of body size along environmental gradients has been studied for more than 160 years [1]. This topic is currently the focus of intense interest, including in the context of the response of organisms to environmental change [2], and a major challenge is disentangling among (phylo)genetic and ecological factors [3], [ 4].

In endotherms, a positive association of body size with latitude and/or elevation, or a negative association with temperature, the so-called Bergmann's rule [1], has been frequently observed (e.g. [5]). An adaptive thermoregulatory explanation for this pattern was originally proposed by Bergmann [1] for which the area-volume ratio of larger sizes facilitates heat conservation in colder environments. However, it has been suggested that other non-exclusive mechanisms can be involved and even confounded with thermoregulatory mechanisms ([5]–[7] and references therein).

For ectotherms, there is a variety of results involving not only the underlying processes but also the observed patterns at the intraspecific and interspecific levels. In reptiles, different studies including different sources of variability reported contrasting results [8]–[13]. Moreover, body size differences between sexes along geographical gradients may exist [14], but have rarely been considered in reptiles and only over fragments of their ranges [15]. All of these sources of variability can yield contrasting results, and have contributed to the ongoing debate on the definition of Bergmann's rule [16]–[19].

Although latitude can explain geographical patterns at coarse scales, an increasing number of studies have encouraged the use of different environmental predictors directly linked with different mechanistic hypotheses [20]. Most of the potential processes proposed are related to physiology or biotic interactions. In ectotherms, the effects of climate and primary productivity on body size can be linked with thermoregulatory strategies, developmental dynamics, activity duration, and/or food availability. Several of these mechanisms are not mutually exclusive and might be acting jointly (e.g. [7]). Patterns and mechanisms commonly considered in previous studies are:

1. Bergmann's rule [8], [ 11]: Larger sizes facilitate heat conservation in colder environments. This rule predicts a negative association between temperature and size.
2. Converse-Bergmann's rule [8]: Smaller sizes facilitate higher heating rates in colder environments. It is predicted a positive association between temperature and size.
3. Temperature-size rule [21], [ 4]: Growth retardation during developmental stages in colder environments involves prolonged growth periods and a delay in maturation, which results in a larger size at maturity. This rule predicts a negative association between temperature and adult size.
4. Seasonality hypotheses: Potential effects of seasonality have been proposed also for opposite size trends in ectotherms. Higher temperatures and/or lower precipitation may reflect longer periods for growing due to an extended activity [22], [ 23]. This mechanism would predict a positive association of size with temperature and a negative association with precipitation. Alternatively, the opposite trend would be also plausible through the joint effects of seasonality and the temperature-size rule; where reduced activity is associated with higher survival, favouring in turn selection for a delayed maturation at larger sizes [24].
5. Resource rule [25], [ 26]: Higher abundance and/or quality of prey during the growing season allow attaining larger sizes, and predict a positive association between primary productivity and size. Also, potential joint effects of productivity and seasonality on body size have been considered for ectotherms [11], [ 27].

Whatever the mechanisms involved, several studies on the geographical body size variation suggested that different possibilities should be considered according to the idiosyncrasies of species [11], [28].

On the other hand, while studies at the inter-specific level often provide analyses accounting for phylogenetic relationships [8], geographical studies of body size variation accounting for phylogenetic relationships among populations are scarce [9], [ 28] and are lacking at the individual level. Indeed, population-level studies in a phylogenetically explicit context may provide information on different evolutionary scenarios. Most phylogenetic studies use techniques to control for phylogenetic autocorrelation [29], [ 8], [ 9], whereas the application of variation partitioning methods to disentangle the independent contributions of ecology and phylogeny from phylogenetic niche conservatism has been only applied to higher taxonomic levels [6]. To our knowledge, there is no study of reptiles on geographical variation in body size that tests for different mechanistic hypotheses while simultaneously accounting for spatial, temporal and phylogenetic autocorrelation for each sex and at the individual level.

In this study, we collected field data and assessed the role of different sources of body size variation in males and females of the Spanish Sand Racer species group. A main challenge in studies of body size variation in vertebrates is accounting for the influences of past inter-annual fluctuations of environmental factors experienced through their entire lives. Thus, the longer their life span the more complex is their trajectory to attain such sizes. The rapid life cycle of sand racers makes them a suitable model to account for short-term temporal variation in body size because most of the environmental influences occur within one year, and age variability of adults is very small. Firstly, we performed a simple ecological model including climatic, topographic and primary productivity predictors that may be linked with previously proposed mechanisms involved in body size variation along gradients. Additional analyses were performed using available bibliographic data and two additional sampled populations, encompassing a time interval of 95 years, to assess the generality of our field-based data. Then, to distinguish between independent and shared effects of ecology and phylogeny [6], we applied a variation-partitioning methodology including ecological variables and pairwise genetic distances at the individual level. Our final models included relevant ecological predictors, while incorporating phylogenetic, spatial and temporal autocorrelation. The main objective of this study was to examine whether alternative sources of variation can emerge only after disentangling and accounting for the independent and shared components of ecology and phylogeny.

Spanish Sand Racers are thermophilic lizards whose distribution encompasses high environmental heterogeneity within the Mediterranean part of the Iberian Peninsula (absent from northern Spain) and southern France: (latitudinal range: 36°0′15″-44°32′6″, longitudinal range: 9°29′54″-6°51′00″). Within Lacertidae they belong to the lizards with smaller size, shorter life span (1–2 years), and earlier sexual maturity (5–9 months, no subadult stage) [30]–[32]. Females are larger than males and they have 1–2 clutches per year [30], [ 31], [ 33]–[35]. Until 2012, Spanish Sand Racers were considered as belonging to one single species with some morphological differentiation among populations. In 2012 three major lineages have been described based on phylogenetic analyses. These lineages diverged 4.8 and 8.3 Mya, and differ in morphology and realised ecological niche [36]. Recently they have been elevated to the species level (*Psammodromus hispanicus*, *P. occidentalis* and *P. edwardsianus*) [37], [ 38].

Considering the above-mentioned main hypotheses without joint effects, several predictions can be made for sand racers. Under Bergmann's and the temperature-size rules we predicted a negative association between temperature and adult body size. Under the seasonality hypothesis we predicted a positive association of body size with temperature, and a negative association with precipitation. Under the resource rule we predicted a positive association between primary productivity and body size.

## Methods

### Field sampling

**Figure 1:**
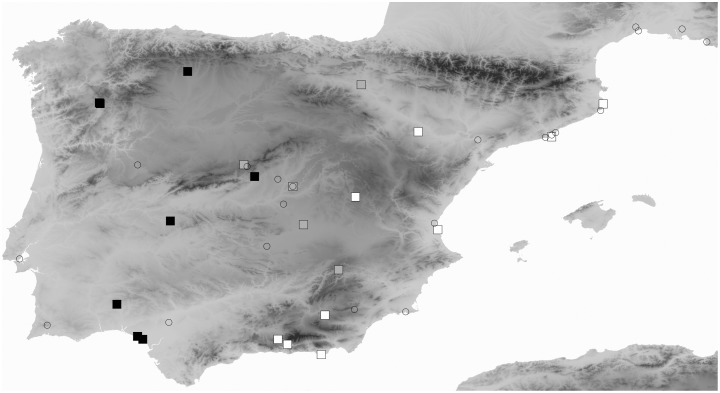
Sampled localities for the Spanish Sand Racer species group corresponding to data-sets obtained from systematic field sampling of mt-DNA and SVL in 2006 (black squares: *Psammodromus occidentalis*, grey squares: *P. hispanicus*, white squares: *P. edwardsianus*) and a review of SVL data in the literature encompassing approximately 100 years (open circles). Higher intensity in the grey scale denotes higher elevation.

Field captures throughout Spain were performed from April to June 2006. Sand racers exhibit a rapid life cycle. They reach sexual maturity in their first spring, there is almost no cohort overlap and juveniles appear mostly in August [31], [ 33]–[35]. During the field-sampling period only adults were observed and captured. Sampling was performed approximately each 250 km within the species distributions (Fig. 1). Environmental variation of sampled populations ranged from 0 to 1355 m a.s.l. for elevation, from 9.4 to 18°C for annual mean temperature, from 246 to 998 mm for annual precipitation and covering a maximum distance of 1005 km. Thus, our sampled populations were representative of the high environmental heterogeneity associated to the species distributions [33]–[35]. Lizard size (snout-to-vent length, SVL) was measured to the nearest 1 mm immediately after capture, and a small piece of the tail tip was collected and preserved in 70% ethanol at −20°C for DNA extraction. We obtained SVL and genetic information for 240 captured adult individuals (130 males and 110 females) from 22 locations; for another 32 captured individuals only genetic information was obtained (see [36] for more details).

Captures and handling of sand racers were accomplished under the permits of Junta de Andalucía, Gobierno de Aragón, Junta de Castilla y León, Junta de Comunidades de Castilla-La Mancha, Generalitat de Catalunya, Junta de Extremadura, Xunta de Galicia, Comunidad de Madrid, Gobierno de Navarra, Generalitat Valenciana, Parque Natural de l'Albufera (Valencia), Parque Natural del Delta del Ebro (Cataluña), Parque Nacional de Doñana (Huelva), and Gobierno de España.

### Genetic distances among individuals

Previous phylogenetic and population genetic studies based on partial sequences of two mitochondrial genes (*cytb*, *nad4*), one nuclear gene (suppressor of SWI4 1) and an unknown nuclear region (clone17) showed congruency of derived phylogenetic trees, i.e., the cytochrome *b* (*cytb*) tree was congruent with the mitochondrial and the nuclear trees and the nuclear trees were as well congruent [36]. Mitochondrial *cytb* sequence data of a 249 bp alignment of partial *cytb* are available from GenBank for all sampled individuals, and sequence data for the other three markers are only available for a small subset [36]. For these reasons, we used the existing Maximum Likelihood phylogenetic tree of *cytb* [36], and calculated cophenetic genetic distances among individuals using the cophenetic function included in the ape package in R 2.7.0 software (Free Software Foundation, GNU Project, Boston, MA, USA). The derived pairwise genetic distance matrix was then used to investigate phylogenetic constraints on geographical body size variation. For additional details concerning the molecular methods used, see Fitze et al. [36].

### Ecological models

We used eight representative ecological predictors: two predictors were related to temperature, two predictors to precipitation, two to plant productivity, one to topography and one with the course of the season. One topographical and five bioclimatic predictors were obtained from the WorldClim source ([39], www.worldclim.org) at 1 km^2^ resolution, and consisted of mean temperature of warmest quarter, mean temperature of coldest quarter, precipitation of wettest quarter, precipitation of driest quarter and mean elevation. As a measure of primary productivity, we used monthly maps of Enhanced Vegetation Indexes (EVI) generated from Moderate Resolution Imaging Spectroradiometer http://modis.gsfc.nasa.gov/(MODIS) satellite images available at the NASA-LP DAAC web page (https://lpdaac.usgs.gov/). We generated year-averaged monthly values of EVI for each grid-cell from the oldest period available to the sampled year (2000–2006). We then calculated the mean EVI for summer and winter for each cell. Thus, among the available temperature, precipitation and productivity predictors we used those encompassing the annual variation. Although the use of environmental predictors directly linked with processes is encouraged, at least one of the traditionally used pattern predictors (latitude or elevation) may be useful for comparisons with other studies (*s. l.* [20]). For this study elevation was included in models because preliminary analyses showed that it significantly correlated with SVL, which was not the case for latitude (see Appendix S1 for a more detailed justification). Finally, to estimate the effect of time on growth in these short-lived lizards with rapid growth, and to explicitly incorporate temporal autocorrelation, we used the capture date as an indicator of the course of the season.

To avoid multicollinearity or redundancy in our analyses, we performed a principal component analysis (PCA, [40]) and obtained eight orthogonal principal components as potential predictors (PC). Multiple regression analyses were performed for males and females with SVL as the dependent variable and the eight PCs as covariates. Significant PCs were considered relevant for subsequent analyses. To further understand the potential role of each ecological predictor, we performed regression analyses for each predictor having a factor loading >0.7 in relevant PCs. Lastly, to understand whether significant predictors reflected one-year or general trends, we used an independent inter-annual data set encompassing 95 years and correlated the environmental predictors that were significant in the intra-annual data set with body size (see Appendix S2).

### Phylogenetic models

To estimate potential phylogenetic inertia on body size, we applied Phylogenetic eigenVector Regression (PVR, [29]) on SVL. We used a double-centred phylogenetic pairwise distance matrix among individuals to perform a Principal Coordinate Analysis (PCoA, [41]) and obtain independent phylogenetic eigenvectors (PV). PCoA yields independent phylogenetic eigenvectors (PV) in an analogous manner as the PCA yields PCs for the ecological models. Thus, the obtained orthogonal PVs can be used as potential predictors of SVL in a regression frame work. This procedure has been shown to be a powerful tool for developing phylogenetically explicit models of body size gradients at higher taxonomic levels [29], [ 3]. An advantage of PVR is that PVs obtained from our individual-based genetic distance matrix can encompass genetic influences at different hierarchical levels (species, clades, sub-clades, populations and individuals), which is more appropriate than exclusively including the species level as a categorical factor in the analyses (see Appendix S3 for statistical tests). The number of derived PVs was large (240), and we used three previously considered selection criteria [6], [ 42]. First, a pre-selection of PVs was based on a descending sequential criterion regarding the cumulative variance explained up to 99%, which yielded the first 30 PVs. Then, multiple regression analyses were performed for males and females with SVL as the dependent variable, to test the significance of these remaining 30 PVs (full models and backward selection yielded same results). Lastly, to further examine phylogenetic autocorrelation, residuals of the final ecological, phylogenetic and combined models were regressed on the 30 retained PVs. This procedure is analogous to that proposed by Bini et al. [43]. Significant PVs in at least one of the analyses were considered relevant for subsequent analyses.

### Disentangling independent and shared components of ecology and phylogeny

To quantify the variance explained by independent components of ecology and phylogeny and by the component shared between them (the phylogenetically structured environmental variation), we used the variation-partitioning method proposed by Desdevises et al. [6], which is analogous to the widely applied general procedure of variation partitioning [44], [ 45]. The variation explained (*R^2^*) by either the ecological or the phylogenetic model is subtracted from the combined model (including both relevant PCs and PVs) to obtain the independent components.

To estimate the significance of the independent components, we followed two steps: 1) extracting the structure not shared between phylogeny and ecology into independent components from the residuals resulting from regressing each relevant PC (dependent variable) on all relevant PVs (independent variables) and each relevant PV on all relevant PCs (hereafter ResPC and ResPV, respectively) and 2) estimating the significance of the independent components obtained in step 1 by performing multiple regressions of SVL on either all ResPC (independent components of ecology) or all ResPV (independent components of phylogeny). See Desdevises et al. [6] for a more detailed description of the overall procedure. Additionally, if a significant ResPC was obtained, residuals were also directly derived for ecological predictors with factor loadings >0.7 (Table 1) in the corresponding PC and tested for significance.

**Table 1:** Principal components analysis for environmental predictors in Peninsular Spain at a 1^2^-resolution.

Ecological predictors	PC-1	PC-2	PC-3	PC-4	PC-5	PC-6	PC-7	PC-8
**Course of Season**	−0.55	0.22	−0.25	**0.72**	0.24	0.09	0.01	0.02
**Mean Temperature of Warmest Quarter**	**0.92**	0.24	0.12	0.15	−0.04	0.16	−0.20	−0.01
**Mean Temperature of Coldest Quarter**	**0.83**	0.51	0.16	0.03	0.02	0.01	0.09	0.13
**Precipitation of Wettest Quarter**	−0.38	**0.81**	−0.09	0.14	−0.30	−0.28	−0.05	−0.01
**Precipitation of Driest Quarter**	−0.49	0.51	−0.42	−0.50	0.26	0.06	−0.07	0.04
**Elevation**	**−0.71**	−0.65	0.02	0.03	−0.20	−0.00	−0.09	0.12
**Enhanced Vegetation Index of Winter**	−0.57	0.17	**0.74**	−0.03	0.28	−0.11	−0.05	0.00
**Enhanced Vegetation Index of Summer**	**−0.74**	0.46	0.25	−0.07	−0.26	0.33	0.05	−0.01
**Eigenvalue**	3.59	1.95	0.90	0.82	0.40	0.24	0.07	0.03
**Variance explained (%)**	44.84	24.41	11.29	10.21	5.06	2.96	0.83	0.40
**Cumulative Variance (%)**	44.84	69.26	80.54	90.76	95.82	98.77	99.60	100

Bold lettering denotes factor loadings >0.7.

Although the shared component of phylogeny and ecology is potentially very important from an evolutionary perspective (reflecting phylogenetic niche conservatism), its significance cannot be estimated directly [6]. However, an approximation controlling for relevant independent effects should be at least informative. In previous studies it has been proposed that the pure effect of the environment can be obtained from the error term when regressing body size on the relevant PVs [29]. We followed the same philosophy but with a variant procedure to control for the relevant independent effects. We regressed SVL only on those independent components that were significant in the above-explained step 2 (*i.e.*, ResPCs and ResPVs expressing the relevant structure not shared between ecology and phylogeny). In this new regression (SVL = ResPV_i_+···+ ResPV_n_ + ResPC_i_ +···+ ResPC_n_ + *e*), the error term *e* represents the new resulting residuals. Thus, the residuals *e* express the remaining variation of SVL that was not explained by the significant independent components. These new residuals *e* were again regressed on all eight PCs and tested for significance (*e* = PC-1 +···+ PC-8). In other words, we estimated the effects of ecological factors on the remaining portion of SVL that was not explained by the unshared variation between ecology and phylogeny. In the same way as for the ecological models, we performed a regression analysis for each ecological predictor with a factor loading >0.7 in significant PCs. Overall, the potential roles of different ecological predictors on the pure and shared effects were inferred through changes in significance from models before and after disentangling the pure and shared effects.

SVL of lizards from field and bibliographic data did not differ significantly from a normal distribution, nor did residuals of phylogenetic and ecological models (Kolmogorov-Smirnov d range = 0.05–0.17, *P*>0.05 in all cases). Specific analyses to reduce dimensionality and assess autocorrelation were performed with R ([46], http://www.r-project.org/), and SAM ([47], http://www.ecoevol.ufg.br/sam/) software.

### Spatial autocorrelation in models

We assessed spatial autocorrelation by adding relevant spatial filters to the ecological, phylogenetic and combined models to estimate the extent to which our predictors absorbed the spatial structure of the SVL data ([47], see Appendix S4 for full details).

## Results

### Ecological models

The first four PCs obtained from the PCA explained 90.8% of the total variance (Table 1). In PC-1, four environmental predictors had factor loadings >0.7, where temperature predictors were positively associated, and elevation and EVI summer were negatively associated. In PC-2, PC-3 and PC-4, precipitation of wettest quarter, EVI winter and course of the season, respectively, had the highest factor loadings, all >0.7 and positive (Table 1).

When including all eight PCs in a multiple linear regression as predictors of SVL, PC-1 was significantly and negatively associated with SVL in both males and females (Table 2). Moreover, significance of PC-1 remained stable upon adding the relevant spatial filters (Table S1 in Appendix S4). However, we found sex differences regarding other PCs. PC-6 was only significant in males, and PC-4 and PC-7 were only significant in females (Table 2). For males, PC-6 remained significant after adding spatial filters with two selection criteria and marginally significant for a third selection criterion. For females, PC-7 was significant for one selection criterion (Table S1 in Appendix S4). Finally, when including only the first four PCs in the analyses, their significance did not change neither for females (PC-1: *b* = −0.29, *t_105_* = −3.18, *P* = 0.002; PC-2: *b* = −0.05, *t_105_ = *−0.60, *P* = 0.552; PC-3: *b* = −0.14, *t_105_ = *−1.54, *P* = 0.126; PC-4: *b* = 0.20, *t_105_ = *2.20, *P* = 0.030), nor for males (PC-1: *b* = −0.26, *t_125_* = −2.96, *P* = 0.004; PC-2: *b* = −0.04, *t_125_ = *−0.42, *P* = 0.675; PC-3: *b* = 0.002, *t_125_ = *0.02, *P* = 0.984; PC-4: *b* = 0.03, *t_125_ = *0.37, *P* = 0.716).

**Table 2:** Multiple linear regression analyses of snout-to-vent-length on the ecological (PC) and phylogenetic (PV) factors, and on independent components, *i.e.* ecology independent of phylogenetic factors (ResPC) and phylogeny independent of ecological factors (ResPV).

	Males			Females		
Ecological factors	*b*	*t (121)*	*P*	*b*	*t (101)*	*P*
**PC-1**	**−0.26**	**−3.04**	**0.002**	**−0.30**	**−3.27**	**0.001**
**PC-2**	−0.03	−0.32	0.747	−0.05	−0.56	0.572
**PC-3**	0.01	0.09	0.926	−0.14	−1.55	0.123
**PC-4**	0.01	0.11	0.908	**0.22**	**2.44**	**0.016**
**PC-5**	−0.03	−0.36	0.720	−0.09	−0.95	0.340
**PC-6**	**−0.20**	**−2.37**	**0.019**	−0.09	−1.02	0.308
**PC-7**	0.12	1.42	0.159	**−0.19**	**−2.06**	**0.042**
**PC-8**	0.11	1.29	0.197	−0.01	−0.12	0.900

Phylogenetic factors	*b*	*t (99)*	*P*	*b*	*t (79)*	*P*
**PV-1**	0.06	0.58	0.559	0.15	1.76	0.081
**PV-2**	**0.30**	**2.00**	**0.048**	**0.32**	**3.32**	**0.001**

Independent components	*b*	*t (127)*	*P*	*b*	*t (106)*	*P*
**ResPC-1**	−0.07	−0.75	0.452	−0.13	−1.34	0.181
**ResPC-4**	-	-	-	**0.19**	**2.04**	**0.043**
**ResPC-6**	**−0.20**	**−2.27**	**0.024**	-	-	-
**ResPC-7**	-	-	-	−0.16	−1.74	0.084

	*b*	*t(128)*	*P*	*b*	*t(108)*	*P*
**ResPV-2**	**0.28**	**3.36**	**0.001**	**0.26**	**2.77**	**0.006**

Bold lettering denotes partial correlations significant at *P* < 0.05. Only the first two phylogenetic factors are shown for brevity, the remaining factors were not significant (See methods for details). *b*: denotes standardized regression coefficients.

**Figure 2:**
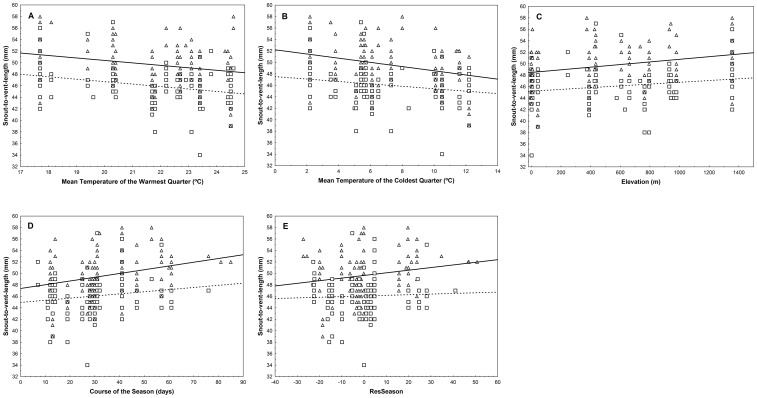
Relationships between snout-to-vent-length of males (squares and dashed line) and females (triangles and solid line) and A) mean temperature of the warmest quarter, B) mean temperature of the coldest quarter, C) elevation, D) course of the season and E) the phylogenetically independent component of the course of the season (ResSeason).

When regressing separately those environmental predictors with factor loadings >0.7 in the significant PCs, we found similarities and differences between sexes (Table 3, Fig. 2). Temperature predictors were significantly and negatively associated with SVL of both males and females (Table 3, Fig. 2a, b). Both sexes also showed a significant positive association of their SVL with elevation (Table 3, Fig. 2c). There were no significant associations with primary productivity in either sex. Interestingly, the course of the season was significantly associated only for females (Table 3, Fig. 2d). Lastly, the analyses performed with the independent 95-year data-set confirmed the observed effects of temperature and elevation (see Appendix S2).

**Table 3:** Linear regression analyses of snout-to-vent-length on each environmental predictor exhibiting factors loadings > 0.7 in significant PCs (see Table 2).

	Males			Females		
Predictors	*F (1,128)*	*b*	*P*	*F (1,108)*	*b*	*P*
**Mean Temperature of Warmest Quarter**	**12.31**	**−0.30**	**< 0.001**	**5.67**	**−0.22**	**0.02**
**Mean Temperature of Coldest Quarter**	**6.15**	**−0.21**	**0.01**	**9.82**	**−0.29**	**0.002**
**Elevation**	**6.78**	**0.22**	**0.01**	**8.15**	**0.26**	**<0.01**
**Enhanced Vegetation Index of Summer**	1.78	0.12	0.18	1.40	0.11	0.24
**Course of the** **Season**	3.13	0.15	0.08	**9.62**	**0.29**	**0.002**
**Phylogenetically independent predictors**						
**ResSeason**	0.24	0.04	0.62	**4.19**	**0.19**	**0.04**
**ResEVIsummer**	0.25	0.04	0.62	0.73	0.08	0.39

Bold lettering denotes *P*-values<0.05. *b*: denotes standardized regression coefficients.

### Phylogenetic models

When including the first thirty PVs as predictors of SVL, only PV-2 was significant in males and females (Table 2), either in the full or backward eliminated models. When regressing residuals of ecological, phylogenetic and combined models on the 30 PVs, again only the PV-2 remained significant for both sexes. PV-2 as well remained significant in both sexes upon adding spatial filters, independently of the filter selection criteria (Table S1 in Appendix S4).

### Independent and shared components of ecology and phylogeny

**Figure 3:**
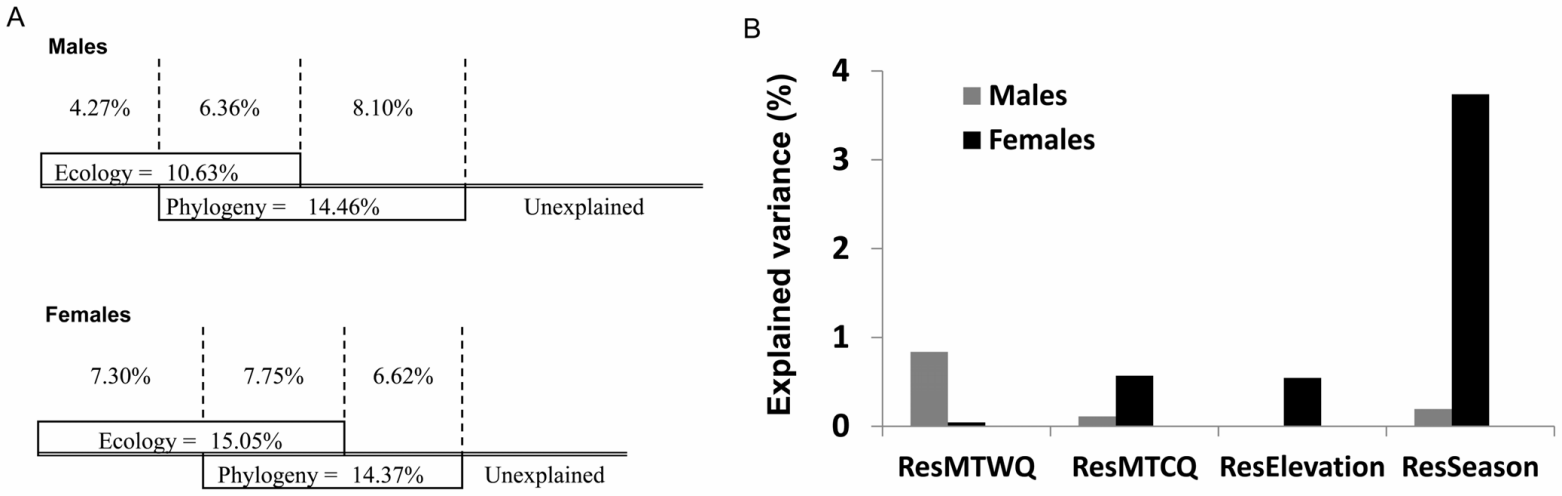
Variation partitioning into different contributions to the snout-to-vent-length of males and females. A) Independent and shared contributions of ecology and phylogeny and B) contributions of the phylogenetically independent component of relevant ecological predictors: mean temperature of the warmest quarter (ResMTWQ), mean temperature of the coldest quarter (ResMTCQ), elevation (ResElevation) and course of the season (ResSeason). Males are depicted in grey and females in black.

Before the partition, the variation explained by phylogeny was similar in both sexes, whereas the variation explained by ecology was greater in females (Fig. 3a). The final combined models explained 19% and 22% of the variance for males and females, respectively. Variation partitioning again revealed similarities and differences between sexes. The independent component of phylogeny and the overlap between ecology and phylogeny were comparable in magnitude between sexes. The absolute difference between the independent phylogeny and the overlap was 1.74% and 1.13% for males and females, respectively (Fig. 3a). In contrast, the independent component of ecology was larger in females than in males (difference: 3.03%, Fig. 3a).

The independent contribution of phylogeny to SVL was significant in both sexes (males: *F_1,128_* = 11.29, *P*<0.002; females: *F_1,108_* = 7.65, *P*<0.007) and remained reasonably stable after adding spatial filters with different selection criteria (ResPV-2, Table S1 in Appendix S4). The independent effect of ecology was significant for females (*F_3,106_* = 2.78, *P*<0.044) but did not reach significance in males (*F_2,127_* = 2.83, P = 0.062). For females, only ResPC-4 (reflecting the variance in PC-4 that could not be explained by phylogeny) showed significance; ResPC-1 did not (Table 2). ResPC-4 was also significant in a regression upon adding the spatial filters with Moran's *I* >0.1 (Table S1 in Appendix S4). The course of the season clearly showed the highest factor loading in PC-4 (Table 1). For this reason, we went directly to the season predictor, controlled for phylogeny (hereafter ResSeason) and included as a predictor of SVL. ResSeason was significantly associated with female SVL but not with male SVL (Table 3, Fig. 2e).

Additionally, the presence of only one relevant PV in the phylogenetic models enabled us to perform variance partitioning of each relevant ecological predictor separately against PV-2 in a balanced way. The independent proportion of explained variation by the course of the season (ResSeason) was much greater in females than in males, and greater than the contribution of the other predictors (Fig. 3b).

**Figure 4:**
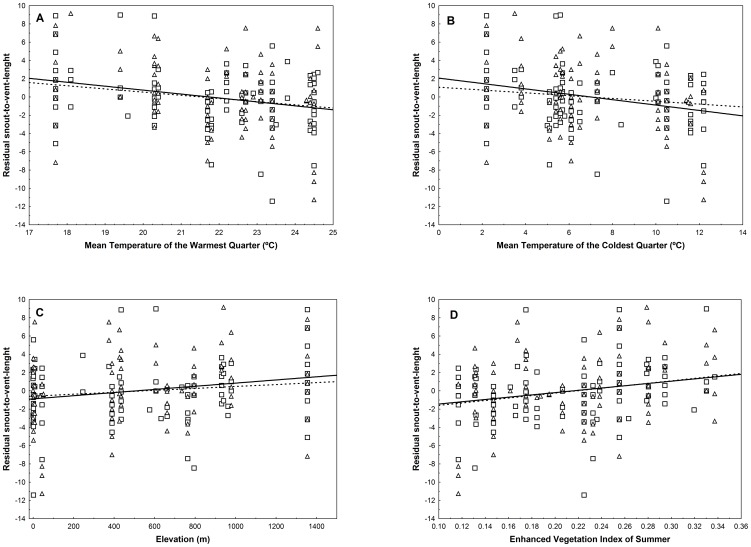
Relationships between the residuals of snout-to-vent-length and A) mean temperature of the warmest quarter, B) mean temperature of the coldest quarter, C) elevation and D) Enhanced Vegetation Index of Summer. Residuals of snout-to-vent-length (denoted as *e* in the formulation of methods) express the portion of SVL that was not explained by the unshared variation between ecology and phylogeny. Squares and dashed line: males, triangles and solid line: females.

To extract the portion of SVL that was not explained by the overlap between ecology and phylogeny, we took the error term *e* from the regressions including all the significant non-shared structures (males: SVL = ResPV2 + ResPC-6 + *e*; females: SVL = ResPV2 + ResPC-4 + *e*, see methods). These error terms *e* (expressed as newly obtained residuals, hereafter residual SVL) were again regressed against all eight PCs. Estimations again revealed significant effects of PC-1 for males and females, remaining significant with the second and fourth filter selection criteria, respectively (Table 4). Also, there was a significant effect of the PC-7 only for females, which remained significant with three filter selection criteria (Table 4). In contrast to the analyses run before separating independent from shared components (Table 2), PC-4 and PC-6 were not significant (Table 4). When regressing separately those environmental predictors with factor loadings >0.7 in significant PCs, the mean temperature of the warmest quarter was negatively and significantly associated with the residual SVL regardless of sex (Table 5, Fig. 4a). Mean temperature of the coldest quarter was negative and significant in females, and a trend was found in males (Table 5, Fig. 4b). Elevation showed significant positive relationships only for females (Table 5, Fig. 4b). An interesting difference from the previous analyses using the entire variation (before separating independent from shared components, Table 3) is that there were positive significant associations of EVI of summer with the residual SVL for both sexes (Table 5, Fig. 4d).

**Table 4:** Multiple regression analyses of the residuals of snout-to-vent-length on the eight ecological factors (PC).

	Males			Females		
Ecological factors	*b*	*t (121)*	*P*	*b*	*t (101)*	*P*
**PC-1**	**−0.28**	**−3.3**	0.001**	**−0.31**	**−3.43**	<0.001****
**PC-2**	0.10	1.2	0.23	0.06	0.64	0.525
**PC-3**	0.02	0.2	0.83	−0.12	−1.27	0.206
**PC-4**	0.02	0.3	0.79	0.02	0.25	0.805
**PC-5**	−0.09	−1.0	0.32	−0.16	−1.77	0.079
**PC-6**	−0.00	−0.0	0.97	−0.10	−1.07	0.288
**PC-7**	0.15	1.8	0.08	**−0.21**	**−2.24**	0.027***
**PC-8**	0.10	1.2	0.23	−0.01	−0.15	0.878

Residuals of snout-to-vent-length (denoted as *e* in the formulation of methods) express the portion of SVL that was not explained by the unshared variation between ecology and phylogeny. Bold lettering denotes partial correlations significant at *P*<0.05. Asterisks denote the number of times that a PC was significant when applying the four selection criteria used to add spatial filters. See methods, Tables 2, 3 and Appendix S4 for details. *b*: denotes standardized regression coefficients.

**Table 5:** Linear regression analyses of the residuals of snout-to-vent-length on each environmental predictor exhibiting factor loadings >0.7 in significant PCs (see Table 4).

	Males			Females		
Environmental predictors	*F (1,128)*	*b*	*P*	*F (1,108)*	*b*	*P*
**Mean Temperature of Warmest Quarter**	**9.20**	**−0.25**	**0.003**	**6.50**	**−0.24**	**0.012**
**Mean Temperature of Coldest Quarter**	3.65	−0.16	0.058	**6.97**	**−0.24**	**0.009**
**Elevation**	3.51	0.16	0.063	**5.04**	**0.21**	**0.026**
**Enhanced Vegetation Index of Summer**	**10.32**	**0.27**	**0.002**	**4.94**	**0.21**	**0.028**

Residuals of snout-to-vent-length (denoted as *e* in the formulation of methods) express the portion of SVL that was not explained by the unshared variation between ecology and phylogeny. Bold lettering denotes *P*-values<0.05. See methods for details. *b*: denotes standardized regression coefficients.

### Spatial autocorrelation in combined models

The spatial structure not absorbed by the combined final models (including the relevant PCs and PVs) was small and did not affect the contribution of predictors in the combined models. Accordingly, the variance explained jointly by ecology and phylogeny independently of the spatial structure was much greater than that explained by the independent spatial component (see Appendix S4).

## Discussion

The two independent data sets analysed separately (the one-year field sampling and the inter-annual one) showed the same pattern and concomitant with Bergmann's cline: SVL increased with elevation and decreased with temperature both in males and females. Our procedures for separating the different sources of SVL variation revealed both similarities and differences between sexes. After separating the shared and independent components of phylogeny and ecology, we found a significant effect of phylogeny (independently of contemporary ecological factors). On the other hand, the effect of the independent component of ecology was much stronger in females than in males because females were much more influenced by the course of the season. Lastly, an effect of primary productivity on SVL in both sexes was only detected after controlling for the independent relevant components of ecology and phylogeny. Precipitation was not significant in any analyses. The fact that our models remained relatively stable after accounting for spatial autocorrelation shows that the predictors used are sufficiently informative to absorb most of the spatial structure of size variation at this scale.

The procedures used here to separate the independent and shared effects of phylogeny and ecology have been previously applied to higher taxonomic levels (the family level) in endotherms [6]. In this study, we applied this methodology using, for the first time, a matrix of genetic pair-wise distances at the individual level. The independent effect of ecology was stronger in females, primarily due to a differential effect of the course of the season, reflecting females grow faster. In agreement, longitudinal recapture data suggested that *P. hispanicus* adult females grew faster than males [30]. In organisms with rapid life cycles (early maturation and short adult lives), such as the species studied here [32], rapid female growth is advantageous, because reproductive success may depend on female body size (e.g., clutch size: [34], [ 35]; production of a second clutch: [31]; mass invested per clutch, or minimum size for producing a clutch). These sex differences could not be tested in the 95-year data-set because information on the course of the season through capture dates was not always available, indicating that sex-differences in developmental processes may be missed if a given dimension is lacking.

The independent effect of phylogeny was similar for males and females, reflecting a phylogenetic inertia that shaped part of the variation in lizard size as has been shown at other phylogenetic scales in endotherms (e.g. [29]). Phylogenetic inertia is the consequence of past evolutionary processes and may include geographic isolation, which has been proposed as an important driver of speciation in sand racers [36].

The proportion of variation explained by the overlap between ecology and phylogeny is similar in magnitude to that of the independent components (Fig. 3). This result is compatible with the existence of a link between contemporary environment and evolutionary mechanisms shaping body size. Once controlled for the relevant independent components, we found the same effect of temperature (Table 4) as when analyzing raw data (Table 1). Interestingly, an additional predictor that was previously overridden was then revealed as a factor with an important role in explaining this form of residual body size. Both sexes showed a significant positive relationship between the residual size and the vegetation index for summer, which reflects primary productivity during the activity period. The fact that PC-4 (in which the factor loading of the course of the season is larger) was no longer significant after controlling for the independent components supports the effectiveness of this procedure in controlling for short-term temporal effects.

Although the course of the season was mainly expressed by a PC factor different from that associated with other significant predictors, other predictors were correlated among them, as expressed in PC-1 (Table 1). Taken together several potential non-exclusive mechanisms on the partial role of the contemporary environment can be proposed in the light of previous studies and the species idiosyncrasy. While the thermoregulatory mechanism originally proposed to explain Bergmann's rule has been argued for endotherms, its role in ectotherm size is more controversial [16], [ 11]. For ectotherms, it is important to distinguish whether clinal variation may exist due to the benefits of larger sizes that are associated with higher thermal inertia or due to the benefits of smaller sizes that are associated with faster heat gain and loss. Although Bergmann emphasised the benefits of larger size in colder environments for endotherms, Kearney et al. [48] showed that the thermal challenge for terrestrial ectothermic animals may be to remain cool, depending on the inhabited climatic niche. From the available information on the climatic niche, morphology, behavior and physiology of sand racers it can be argued that, if a thermoregulatory mechanism was involved in the geographical pattern, it should be more related to heat loss in relatively warmer places. The Mediterranean climatic range of sand racers is warmer than that of other northern or montane distributions of European lacertids. Phylogenetically independent contrast of a lacertid clade reported that smaller species heat faster. Thus, sand racers gained heat most rapidly due to their relatively small size, which should be a constraint when operative ambient temperatures are above the preferred range [49]. Once corrected by size authors inferred an adaptive physiological mechanism to reduce overheating in Mediterranean lizards. Moreover, thermoregulatory behavioural strategies for gaining heat are less pronounced in Mediterranean lacertids (including *Psammodromus*) than in other northern/montane European lacertids [49]. This scenario suggests that the thermal challenge for smaller individuals is more strongly associated with the avoidance of overheating. Therefore, the observed geographical pattern (smaller lizards in warmer places) would not match a thermoregulatory mechanism in a simple manner, but rather through a complex syndrome linking different aspects of the species biology as in other Mediterranean lacertids [50].

Several alternatives to thermoregulatory mechanisms have been proposed for ectothermic animals. The so-called temperature-size rule proposes that ectothermic animals in hotter environments grow faster but, paradoxically, mature at a smaller body size [21], [ 51]. A single explanation for the generality of this rule is lacking [52]. An explanation of the temperature-size rule for the lizard *Sceloporus undulatus* is that juveniles in colder environments have higher survival, mediated by biotic interactions, allowing them to delay maturation until they attain larger sizes, which provides a reproductive advantage [9]. Data on size at maturity are scarce in sand racers and restricted to females, but anecdotal evidence is consistent with the mechanism proposed by Angilletta et al. [9]: in Salamanca, the mean temperature of warmest quarter (21.1°C) is lower and the size at maturity (42 mm) and the mean adult size (48 mm) are higher [30] than in Barcelona (23.4°C, 38.7 mm and 42.8 mm, respectively, [53]). In *Z. vivipara*, a lacertid which inhabits a much larger geographic area, the temperature-size rule in combination with an effect of annual activity has been suggested to affect body size: reduced activity in colder environments has been suggested to increase survival and favour larger sizes at maturity [25]. Alternatively, it has been argued that in colder and seasonal environments larger sizes would be advantageous to survive periods of food shortage by means of fat storage [24]. In the Iberian Peninsula colder environments are often associated with a greater seasonality, and hence an effect of activity duration might partially shape sand racer sizes. However, this cannot be the single factor influencing body sizes since it is known that precipitation precludes activity in lacertid species [54], and we did not find any effect of precipitation in our analyses.

Once controlled by relevant independent effects, body size was positively correlated with primary productivity with the highest relative contribution (Table 5). The finding that the vegetation index of summer (when juveniles hatch), but not that of winter, predicted body size indicates that primary productivity during the activity period is an important determinant of size. Plant productivity is an indicator of invertebrate prey productivity [27]. An obvious direct effect of food availability (quantity or quality of prey) on lizard size is through fitness components, such as growth or survival, which is crucial at the juvenile stage in lacertids [55]. In this line, taking also into account additional analyses with a surrogate of lizard density (see Appendix S3) it appears that higher primary productivity allows a greater lizard biomass (greater number of lizards and larger sizes).

On the other hand, in this study the effect of primary productivity emerged only after correcting for phylogenetic inertia and short-term temporal variation within the season. This result invites to consider as plausible the role of contemporary environmental factors in evolutionary scenarios, besides the evident effect of food abundance on postnatal growth. A previous study suggested that prey size is an important driver of the evolution of size at birth in tiger snakes because it is correlated with gape size, which ultimately affects adult size in combination with the effect of resource availability during ontogeny [56]. A translocation of the lacertid, *Podarcis sicula*, into a novel environment revealed an evolutionary divergence in morphology due to strong changes in the diet [57]. Thus, it has been suggested that the temperature-size relationships may act through food intake during the growing season [27], in combination with the pulse of seasonality [7], or in combination with a direct effect of temperature [51]. The latter scenario might partially conciliate the puzzle in the temperature-size rule for ectothermic terrestrial vertebrates; in which many ectotherms grow more slowly but mature at a larger size at lower temperatures. Thus, higher juvenile survival due to higher food availability might outweigh the costs of delayed maturation [51], or higher growth rates due to a higher food intake might counteract growth retardation at lower temperatures.

## Conclusions

Our one year-field work and the multi-year data base converged showing that lizard size is negatively associated with temperature, concomitantly with Bergmann's and temperature-size rules. The applied procedures to disentangle among independent and shared components of ecology and phylogeny allowed detecting sources of variation connecting with different non-exclusive mechanisms. These procedures showed independent effects of phylogenetic inertia and sex-dependent short-term temporal autocorrelation. Primary productivity only emerged as an important predictor of size after controlling for phylogenetic inertia and sex-differences in growth, and consistently with the resource rule. This study highlights the importance of including individual-based genetic information, informative ecological parameters of different nature, and temporal and spatial autocorrelation in sex-specific models to detect potentially important hidden effects. Finally, besides the use of procedures to disentangle inter-correlations among environmental predictors, we encourage focusing on the species' idiosyncrasies to interpret patterns of body size geographical variation.

## Supporting Information

Appendix S1 **Additional analyses with latitude and initial considerations.**
(DOC)

Appendix S2 **Evaluating the generality of patterns with an independent inter-annual data-set.**
(DOC)

Appendix S3 **Additional analyses to further exploring potential mechanisms.**
(DOCX)

Appendix S4 **Spatial autocorrelation in models.**
(DOC)
