# Supplementary material for: Geographical and Temporal Body Size Variation in a Reptile: Roles of Sex, Ecology, Phylogeny and Ecology Structured in Phylogeny
Source: PLoS One. 2014 Aug 4;9(8):e104026. doi: 10.1371/journal.pone.0104026 (PMC4121295; doi:10.1371/journal.pone.0104026)
Supplement: Appendix S1 — Additional analyses with latitude and initial considerations. (DOC) [file pone.0104026.s001.doc]

**Appendix S1: Additional analyses with latitude and initial considerations**

We performed preliminary analyses with latitude and elevation separately to decide which one is preferable including in models. We performed simple regression analyses, with body size as the dependent variable and either elevation or latitude as the predictor. There was a significant positive association of body size with elevation regardless of sex (males: *F_1,128_* = 6.79, *R²* = 0.05, *P* = 0.01; females: *F_1,108_* = 8.15, *R²* = 0.07, *P* = 0.005), but there was no significant association of body size with latitude (males: *F_1,128_* = 0.86, *R²* = 0.006, *P* < 0.35; females: *F_1,108_* = 0.20, *R²* = 0.001, *P* < 0.65). Thus, our results differ from other studies at coarser scales in that latitude was not significantly associated with size (e.g. Ashton & Feldman 2003, Cruz *et al*., 2005). On the Iberian Peninsula, the temperature gradient from the coast to inland areas in combination with elevation heterogeneity, indicates that latitude does not express the full complexity of temperature gradients at our spatial resolution. Therefore, our results support previous studies that encourage the use of direct predictors rather than *a priori* assumed surrogate variables (Stillwell 2010).

We therefore used elevation because it is a commonly used pattern predictor that is comparable across studies and that might also reflect unexpected mechanisms, such as anthropogenic disturbance (Nogués-Bravo *et al*., 2008). Furthermore, models were tested with relevant spatial filters to examine whether other spatial structures, in addition to latitude, were explained by the predictors used (see Spatial Autocorrelation section).

Given these initial considerations, ecological models were built including temperature, precipitation, vegetation indexes, elevation and the course of the season. We used procedures to generate orthogonal predictors of ecology, phylogeny, temporal and spatial structure and to reduce dimensionality in both the ecological and the phylogenetic models.

**References**

Ashton KG, Feldman CR (2003) Bergmann's rule in nonavian reptiles: turtles follow it, lizards and snakes reverse it. Evolution 57: 1151-1163.

Cruz FB, Fitzgerald LA, Espinoza RE, Schulte II JA (2005) The importance of phylogenetic scale in tests of Bergmann’s and Rapoport’s rules: lessons from a clade of South American lizards. J Evol Biol 18: 1559-1574.

Nogués-Bravo D, Araujo MB, Romdal T, Rahbek C (2008) Scale effects and human impact on the elevational species richness gradients. Nature **453**: 216-U8.

Stillwell RC (2010) Are latitudinal clines in body size adaptive? Oikos 119: 1387-1390.
