# Supplementary material for: Geographical and Temporal Body Size Variation in a Reptile: Roles of Sex, Ecology, Phylogeny and Ecology Structured in Phylogeny
Source: PLoS One. 2014 Aug 4;9(8):e104026. doi: 10.1371/journal.pone.0104026 (PMC4121295; doi:10.1371/journal.pone.0104026)
Supplement: Appendix S2 — Evaluating the generality of patterns with an independent inter-annual data-set. (DOC) [file pone.0104026.s002.doc]

**Appendix S2: Evaluating the generality of patterns with an independent inter-annual data-set**

To understand whether the derived predictions were of general validity or restricted to the sampling period, we performed an exhaustive literature search and compiled all available Spanish Sand Racer SVL measurements whenever sex and capture coordinates (or precise locality) were reported. We obtained SVL data for 20 geographical locations in Spain, France and Portugal (Boulenger, 1921; Mertens, 1925; Pascual & Pérez-Mellado, 1989; Carretero, 1994; López & Martín, 2009; Fitze *et al*., 2012; see Fig. 1) and added data collected in two additional populations in 2012 (40º 27' 22.14'', 4º 10' 28.36'' and 39º 52' 23'', 3º 33' 50''). The inter-annual data-set encompassed a 95-years time interval and consisted of averages per sex for three locations from three publications, and of individual measurements for the rest of locations (three publications and data collected in 2012). Most of these studies give geo-referenced sampling locations with coordinates, and two of them give precise information on the locality. To avoid bias in the precision of the geographical locations, we used environmental variables at 5 km of resolution for this inter-annual data-set. Environmental predictors that were significant in the intra-annual raw data were used in linear regressions as independent variables to test for significant association with SVL.

These analyses confirmed the results of the intra-annual data (see main text). There were significant negative correlations between SVL and mean temperature of warmest quarter for both males and females (males: *n* = 45, *b* = -0.31, *F* = 4.55, *P* = 0.03; females: *n* = 54, *b* = -0.29, *F* = 4.73, *P* = 0.03; Fig. S1a). SVL was significantly and negatively correlated with mean temperature of coldest quarter (males: *b* = -0.50, *F* = 14.51, *P* = 0.0004; females: *b* = -0.36, *F* = 7.91, *P* = 0.006; Fig. S1b) and the relationship between SVL and elevation was positive and significant for both sexes (males: *b* = 0.51, *F* = 15.10, *P* = 0.0003; females: *b* = 0.51, *F* = 18.59, *P* < 0.0001; Fig. A1c).

**Figure S1.** Relationships between mean snout-to-vent-length of males (squares and dashed line) and females (triangles and solid line) and a) mean temperature of the warmest quarter, b) mean temperature of the coldest quarter, and c) elevation. Filled and open symbols denote means and individual values, respectively.

**References**

Boulenger, G.A. 1921. *Monograph of the Lacertidae, Vol. II.* Longmans, Green & Co. British Museum (Natural History), London.

Carretero MA (1994) Algunes dades morfomètriques de *Psammodromus hispanicus* al Prat de Llobregat. Butll nat delt Llob 1: 13-19.

Fitze PS, González-Jimena V, San-José LM, San Mauro D, Suarez T, Zardoya, R (2012) A new species of sand racer, *Psammodromus* (Squamata: Lacertidae), from the Western Iberian Peninsula. *Zootaxa* 3205: 41-52.

López, P. & Martín, J. 2009. Lipids in femoral gland secretions of mate lizards, *Psammodromus hispanicus*. Biochemical Systematics and Ecology, **37**, 304-307.

Mertens, R. 1925. Amphibien und Reptilien aus dem nördichen und östlichen Spanien. *Abhandlungen herausgegeben von der Senckenbergischen Naturforschenden Gesellschaft*. Neununddreissingster band. Heft 1, pp. 27-129. Senckenbergischen Naturforschenden Gesellschaft, Frankfurt A.M.

Pascual JA, Pérez-Mellado V (1989) Datos sobre la reproducción y el crecimiento de *Psammodromus hispanicus* Fitzinger, 1826 en un medio adehesado de la España Central. Doñ Act Vertebr 16: 45-55.
