# Supplementary material for: Geographical and Temporal Body Size Variation in a Reptile: Roles of Sex, Ecology, Phylogeny and Ecology Structured in Phylogeny
Source: PLoS One. 2014 Aug 4;9(8):e104026. doi: 10.1371/journal.pone.0104026 (PMC4121295; doi:10.1371/journal.pone.0104026)
Supplement: Appendix S3 — Additional analyses to further exploring potential mechanisms. (DOCX) [file pone.0104026.s003.docx]

**Appendix S3: Additional analyses to further exploring potential mechanisms**

**Testing for differences among species explicitly**

Our PVs are generated from a genetic distance matrix at the resolution of the individual level, and therefore has the potential to encompass information on the (phylo)genetic structure at distinct hierarchical levels; ranging in this study from the species, clades, sub-clades, populations, etc., to the individual level. The first PVs tend to represent information on the higher phylogenetic levels, expressing divergences closer to the root of the phylogeny (Diniz-Filho *et al*. 2012). Thus, in our study the first PVs may include information on species and main clade levels and so on through the hierarchical subdivision of branches until the individual level within the last PVs. However, it is not straightforward to know exactly which type of information is associated to a specific PV, precluding interpretations on what type of (phylo)genetic inertia is reflecting the PV2 in our results (see results in the main text). To examine whether the relationships between lizard snout-to-vent length (SVL) and the relevant ecological factor were different among the sand racer species we performed an additional analysis. We performed a General Linear Model (GLM) including SVL as the dependent variable, and as independent variables the species and sex as categorical factors, and the PC-1 and PV2 as continuous predictors. PV2 was included to control for other potential (phylo)genetic effects different of the species level. The initial GLM included all main terms and first-order interactions, and the final model was reached through a backward selection procedure (Quinn and Keough 2002). Lizard SVL was square-root transformed to full-fill the assumptions underlying GLMs; residuals of this GLM were normally distributed (Chi-Square test = 11.41, *P* = 0.12), and variances were homogeneous through the levels of categorical factors and their interactions (Levene's Tests: *F* = 1.53-3.36, *P* > 0.5 in all cases).

According to the sexual size dimorphism in sand racers, there was a significant main effect of sex on SVL (*F_1, 230_* = 69.48, *P* < 0.001). In agreement with the other analyses (see results in the main text) there were significant associations between SVL and the PC-1 (*F_1, 230_* = 4.86, *P* < 0.03) and PV2 (*F_1, 230_* = 8.12, *P* < 0.005) as main effects. There was a significant interaction between the species factor and the PV2 (F*_2, 230_* = 3.60, *P* < 0.03), indicating that the (phylo)genetic inertia differed among species at other levels than at the species level. However, the interaction between the species factor and the PC-1, and all other terms were not significant (*F* range = 0.04-2.18, *P* > 0.12). Thus, the relationship between environmental variables and SVL was not different among sand racer species. Taken together, these results indicate that the (phylo)genetic inertia for SVL found in this study cannot be exclusively associated to the species level alone but also to other (phylo)genetic levels, somewhere within the range from clades to populations or individuals. Therefore, in this study the use of PVR is more appropriate than the simple incorporation of the species as a factor in analyses.

**Testing the role of lizard density**

To explore whether there exists a role of lizard density on SVL we performed an additional GLM. The dependent variable was the residuals of the final models for males and females (see results in main text) as a form of SVL after controlling for the relevant eco-environmental and phylogenetic factors. As a surrogate of density we obtained the number of lizards captured per sampled population. The initial GLM included the sex factor, the density surrogate and their interaction as independent variables, and the final model was reached through a backward selection procedure (Quinn and Keough 2002). There were no significant effects of sex (*F_1, 237_* < 0.001, *P* > 0.99), density (*F_1, 238_* = 0.22, *P* = 0.64) or the interaction between then (*F_1, 236_* = 1.25, *P* = 0.26) on the corrected SVL.

On the other hand, we performed regression analyses to test whether there is a relationship between our surrogate of summer primary productivity (Enhanced Vegetation Index, EVI; see main text) and our surrogate of lizard density. We found that the number of lizards captured per population was positively correlated with EVI of summer (*F_1, 238_* = 58.3, *P* < 0.0001). This result suggests that, sites with higher primary productivity (base of the trophic chain) may allow higher densities, because quantity or quality of food is higher. Given that SVL was positively correlated with primary productivity (see results in the main text, Table 5, Fig. 4d), our results suggest that higher primary productivity allows a greater lizard biomass (greater number of lizards and larger sizes), rather than a pervasive effect of density on size. In agreement, a previous study on Mediterranean lizards in one population, including sand racers, did not find that smaller lizards occur at greater densities but that size was influenced by habitat type and biotic interactions (Gálvez-Bravo *et al*. 2009). This does not necessarily imply that density cannot affect size negatively in other species, because it might depend on the idiosyncrasy of each species. Taken our results together, they suggest that density of sand racers is likely the consequence of environmental effects and in the same direction as SVL, rather than that density could be an additional driver shaping SVL.

**Reference**

Diniz-Filho, J.A.F., Bini, L.M., Rangel, T.F., Morales-Castilla, I., Olalla- Tárraga, M.A., Rodriguez, M.A., Hawkins, B.A. (2012) On the selection of phylogenetic eigenvectors for ecological analysis. Ecography 35: 239-249.

Gálvez-Bravo L., Belliure, J., Rebollo, S. (2009) European rabbits as ecosystem engineers: warrens increase lizard density and diversity. Biodiversity and Conservation 18: 869-885.

Quinn GP, Keough MJ (2002) Experimental Design and data Analysis for Biologist. Cambridge: Cambridge University Press. 537 p.
