# Supplementary material for: Geographical and Temporal Body Size Variation in a Reptile: Roles of Sex, Ecology, Phylogeny and Ecology Structured in Phylogeny
Source: PLoS One. 2014 Aug 4;9(8):e104026. doi: 10.1371/journal.pone.0104026 (PMC4121295; doi:10.1371/journal.pone.0104026)
Supplement: Appendix S4 — Spatial autocorrelation in models. (DOC) [file pone.0104026.s004.doc]

**Appendix S4: Spatial autocorrelation in models**

Latitudinal and longitudinal coordinates of lizard localities were used to perform Principal Coordinates of Neighbor Matrices (PCNM), and to extract positive eigenvector-based spatial filters from a truncated matrix of geographic distances among sampling sites (Rangel *et al*., 2006). Spatial filters were treated as candidate explanatory variables in ecological models, either before or after extracting the pure component (including relevant PCs or ResPCs respectively). Also, spatial filters were added to the phylogenetic models, either before or after extracting the pure component (including relevant PVs or ResPVs respectively). As for phylogenetic vectors, there is not a fixed rule to uncover the relevant spatial eigenvectors to be included in analyses. We followed four previously proposed criteria based on significance, degree of autocorrelation and its minimization (Bini *et al*., 2009). We considered as relevant spatial filters those significantly associated with SVL or with the residuals of models before adding the spatial filters, and those with a Moran’s *I* > 0.1, or with a Moran’s *I* > 0.5 at the first class distance. Models were re-run separately four times, adding each time the subset of spatial filters obtained with each criterion. We examined whether the addition of spatial filters modified the significance and/or coefficients in eco-environmental models and phylogenetic models (Table S2, see also results in the main text).

Finally, we used correlograms to test significance of Moran’s *I* at any distance class in the residuals of combined models (including both relevant PCs and PVs). Spatial filters significantly associated with these residuals were added to the combined models to examine whether PCs and PVs may change their significance and/or coefficients.

The combined final models showed differences between sexes in the spatial autocorrelation of their residuals. For males there were no significant Moran’s *I* at any distance class in the residuals of the combined final model, whereas for females there were significant Moran’s *I* in the 50% of the distance classes. However, these values were not too large (Moran’s *I* positive range: 0.09/0.169; Moran’s *I* negative range: -0.164/-0.107). In fact, only one spatial filter was significantly associated with the residuals of the final combined model of females. Furthermore, once added this spatial filter in the final combined model of females, the results remain the same in partial correlations (PV-2: *b* = 0.23, *t* = 2.32, *P* = 0.022; PC-1: *b* = -0.36, *t* = -2.89, *P* = 0.005; PC-4: *b* = 0.19, *t* = 2.26, *P* = 0.026; PC-7: *b* = -0.23, *t* = -2.61, *P* = 0.01). Accordingly, the variance explained by the final combined model in females (22 %) in comparison with that of the relevant spatial filter (0.07 %) represent an increment by two orders of magnitude.

**References**

Bini LM, Diniz-Filho JAF, Rangel TFLVB, Akre TSB, Albaladejo RG, et al. (2009) Coefficient shifts in geographical ecology: an empirical evaluation of spatial and non-spatial regression. Ecography 32: 193-204.

Rangel TFLVB, Diniz-Filho JAF, Bini LM (2006) Towards an integrated computational tool for spatial analysis in macroecology and biogeography. Glob Ecol Biogeogr 15: 321-327.

**Table S1.** Partial regression standard coefficients (*b*), *t* statistics and associated *P*-values for the significant ecological (PC) and phylogenetic (PV) factors in the ecological and phylogenetic models (see Table 2) for males and females. PCs/PVs and ResPCs/ResPVs refer to models including and excluding the overlap between ecology and phylogeny respectively. Values are shown for ordinary least squares (OLS) models, and for models adding spatial filters (SP) with Moran’s *I* > 0.1 and > 0.5; adding spatial filters significantly associated with SVL (SP-SVL), and adding spatial filters significantly associated with OLS residuals (SP-RES) from either the final phylogenetic or ecological models. Asterisks denote the number of times that each factor remained significant through the four criteria used to add spatial filters.

|  | OLS | | | SP, *I* > 0.1 | | | SP, *I* > 0.5 | | | SP-SVL | | | SP-RES | | |
| --- | --- | --- | --- | --- | --- | --- | --- | --- | --- | --- | --- | --- | --- | --- | --- |
| Ecological models | *b* | *t* | *P* | *b* | *t* | *P* | *b* | *t* | *P* | *b* | *t* | *P* | *b* | *t* | *P* |
| Males |  |  |  |  |  |  |  |  |  |  |  |  |  |  |  |
| PC-1*** | -0.26 | -3.06 | 0.003 | -0.33 | -1.73 | 0.086 | -0.44 | -2.53 | 0.013 | -0.26 | -3.02 | 0.003 | -0.28 | -3.45 | <0.001 |
| PC-6** | -0.20 | -2.41 | 0.017 | -0.30 | -2.06 | 0.042 | -0.23 | -1.93 | 0.056 | -0.18 | -1.61 | 0.109 | -0.21 | -2.68 | 0.008 |
| ResPC-6* | -0.20 | -2.26 | 0.025 | -0.27 | -1.79 | 0.075 | -0.09 | -0.86 | 0.393 | -0.09 | -0.80 | 0.427 | -0.21 | -2.63 | 0.010 |
| Females |  |  |  |  |  |  |  |  |  |  |  |  |  |  |  |
| PC-1*** | -0.29 | -3.22 | 0.002 | -0.45 | -2.42 | 0.017 | -0.41 | -2.20 | 0.03 | -0.14 | -1.31 | 0.193 | -0.497 | -4.69 | <0.001 |
| PC-4 | 0.20 | 2.27 | 0.025 | -0.14 | -0.88 | 0.382 | 0.19 | 1.72 | 0.089 | 0.04 | 0.35 | 0.724 | 0.14 | 1.57 | 0.118 |
| PC-7* | -0.17 | -1.96 | 0.052 | -0.19 | -1.38 | 0.171 | -0.29 | -2.95 | 0.004 | -0.09 | -0.91 | 0.364 | -0.17 | -1.75 | 0.083 |
| ResPC-4* | 0.18 | 1.94 | 0.055 | -0.31 | -2.07 | 0.041 | 0.06 | 0.61 | 0.543 | -0.02 | -0.17 | 0.863 | 0.04 | 0.39 | 0.696 |
|  |  |  |  |  |  |  |  |  |  |  |  |  |  |  |  |
| **Table A2.** (*Continued*) | |  |  |  |  |  |  |  |  |  |  |  |  |  |  |
|  | OLS | | | SP, *I* > 0.1 | | | SP, *I* > 0.5 | | | SP-SVL | | | SP-RES | | |
| Phylogenetic models | *b* | *t* | *P* | *b* | *t* | *P* | *b* | *t* | *P* | *b* | *t* | *P* | *b* | *t* | *P* |
| Males |  |  |  |  |  |  |  |  |  |  |  |  |  |  |  |
| PV-2**** | 0.38 | 4.65 | <0.001 | 0.44 | 2.71 | 0.008 | 0.34 | 3.07 | 0.003 | 0.29 | 3.42 | <0.001 | 0.41 | 4.65 | <0.001 |
| ResPV-2**** | 0.28 | 3.36 | 0.001 | 0.37 | 2.29 | 0.024 | 0.34 | 2.85 | 0.005 | 0.19 | 2.21 | 0.029 | 0.31 | 3.089 | 0.002 |
| Females |  |  |  |  |  |  |  |  |  |  |  |  |  |  |  |
| PV-2**** | 0.38 | 4.26 | <0.001 | 0.34 | 2.62 | 0.01 | 0.40 | 3.05 | 0.003 | 0.22 | 1.99 | 0.050 | 0.36 | 4.13 | <0.001 |
| ResPV-2** | 0.26 | 2.77 | 0.006 | 0.22 | 1.79 | 0.076 | 0.297 | 2.394 | 0.019 | 0.12 | 1.23 | 0.22 | 0.23 | 2.04 | 0.044 |
